# Usefulness of the Primary Tumor Standardized Uptake Value of Iodine-123 Metaiodobenzylguanidine for Predicting Metastatic Potential in Pheochromocytoma and Paraganglioma

**DOI:** 10.1007/s11307-024-01952-8

**Published:** 2024-09-18

**Authors:** Mitsuho Hirahara, Masatoyo Nakajo, Ikumi Kitazano, Megumi Jinguji, Atsushi Tani, Koji Takumi, Kiyohisa Kamimura, Akihide Tanimoto, Takashi Yoshiura

**Affiliations:** 1 Department of Radiology, Graduate School of Medical and Dental Sciences, Kagoshima University, 8-35-1 Sakuragaoka, Kagoshima, 890-8544 Japan; 2 Department of Pathology, Graduate School of Medical and Dental Sciences, Kagoshima University, 8-35-1 Sakuragaoka, Kagoshima, 890-8544 Japan; 3 Department of Radiology, Nanpuh Hospital, 14-3 Nagata, Kagoshima, 892-8512 Japan; 4 Department of Advanced Radiological Imaging, Graduate School of Medical and Dental Sciences, Kagoshima University, 8-35-1 Sakuragaoka, Kagoshima, 890-8544 Japan

**Keywords:** Pheochromocytoma, Paraganglioma, Grading system for adrenal pheochromocytoma and paraganglioma (GAPP), ^123^I-metaiodobenzylguanidine, SUV

## Abstract

**Purpose:**

To examine the usefulness of semi-quantitative analysis using the standardized uptake value (SUV) of iodine-123 metaiodobenzylguanidine ([^123^I]-MIBG) for predicting metastatic potential in patients with pheochromocytoma (PHEO) and paraganglioma (PGL).

**Procedures:**

This study included 18 PHEO and 2 PGL patients. [^123^I]-MIBG visibility and SUV-related parameters (SUVmax, SUVmean, tumor volume of [^123^I]-MIBG uptake [TV_MIBG], and total lesion [^123^I]-MIBG uptake) were compared with the pathological grading obtained using the Pheochromocytoma of the Adrenal Gland Scaled Score (PASS) and the Grading System for Adrenal Pheochromocytoma and Paraganglioma (GAPP), which are used to predict metastatic potential. The PASS scores were categorized as < 4 and ≥ 4. Based on the GAPP scores, PHEOs/PGLs were categorized as follows: well, moderately, and poorly differentiated tumors. The Mann–Whitney U test or Spearman’s rank correlation was used to assess differences or associations between two quantitative variables.

**Results:**

All PHEOs/PGLs were visualized on [^123^I]-MIBG scintigraphy. There were 16 PASS < 4 and 4 PASS ≥ 4 tumors. Moreover, 11 and 9 tumors were well and moderately differentiated, respectively. The uptake scores and SUV-related parameters significantly differed between tumors with a PASS score of < 4 and those with a PASS score of ≥ 4 (each, *p* > 0.05). Moderately differentiated tumors had significantly higher uptake scores and SUV-related parameters except TV_MIBG than well-differentiated tumors (each, *p* < 0.05). The GAPP score was positively correlated with the uptake scores and SUV-related parameters (each, *p* < 0.05) except TV_MIBG.

**Conclusions:**

The primary tumor [^123^I]-MIBG uptake assessed using SUV-related parameters can be an imaging tool for predicting metastatic potential in patients with PHEO/PGL.

**Supplementary Information:**

The online version contains supplementary material available at 10.1007/s11307-024-01952-8.

## Introduction

Pheochromocytomas (PHEOs) and paragangliomas (PGLs) are neuroendocrine tumors originating from the adrenal medulla and extra-adrenal ganglion, respectively. Before the 2017 WHO classification update, PHEOs and PGLs were labeled as either “benign” or “malignant.” The 2017 classification introduced a new perspective, moving away from these terms, and instead focusing on the molecular characteristics to redefine the classification of PHEOs/PGLs [1]. As a result, all PHEOs/PGLs are now regarded as having varying degrees of metastatic potential, much like epithelial neuroendocrine tumors [1]. To predict metastasis, two pathological grading systems, namely, the Pheochromocytoma of the Adrenal Gland Scaled Score (PASS) [2] and the Grading System for Adrenal Pheochromocytoma and Paraganglioma (GAPP), are used [3]. The PASS system comprised 12 individual histologic features with a total score of 20 (range: 1–20). This system has been frequently used to identify metastatic potential in PHEOs/PGLs, and a PASS score of ≥ 4 indicated metastatic behavior or biologically more aggressive tumors [2, 4]. By contrast, the GAPP system with a total score of 10 (range: 0–10) used both histological and clinical parameters including biochemical data and catecholamine type [3]. The GAPP system is utilized to stratify PHEOs/PGLs into three types, which are as follows: well, moderately, and poorly differentiated tumors [3]. Both systems had a high prediction sensitivity of metastatic tumors (90%–100%) [5]. Thus, a noninvasive diagnostic method that can predict the pathological grading of PHEOs/PGLs may be useful for pretreatment risk stratification in patients with PHEOs/PGLs.

Scintigraphy with iodine-123 metaiodobenzylguanidine ([^123^I]-MIBG), a radiolabeled noradrenaline analog, is a good imaging tool for localizing PHEOs/PGLs [6–8]. Metastatic PHEOs/PGLs are more likely to be false-negative than nonmetastatic PHEOs/PGLs on [^123^I]-MIBG scintigraphy [9]. De-differentiation can be associated with [^123^I]-MIBG scintigraphy-negative PHEOs/PGLs [10]. However, metastatic PHEOs/PGLs have a higher [^123^I]-MIBG or [^131^I]-MIBG uptake than nonmetastatic PHEOs/PGLs [11, 12]. Therefore, previous reports have contrasting results about the association between [^123^I]-MIBG uptake and metastatic potential of PHEOs/PGLs.

Recently, semi-quantitative analysis using standardized uptake value (SUV) has been applied in single-photon emission computed tomography (SPECT)/computed tomography (CT) scan [13]. To the best of our knowledge, one study examined the relationship between [^123^I]-MIBG maximum SUV (SUV max) and tumor-to-background ratio in patients with refractory PHEOs/PGLs [14]. However, no study has previously investigated the association between the [^123^I]-MIBG uptake assessed based on SUV and pathological grading in PHEOs/PGLs. Thus, this study aimed to examine whether semi-quantitative analysis using the SUV of tumor [^123^I]-MIBG uptake is useful for characterizing the metastatic potential in patients with PHEOs/PGLs.

## Materials and Methods

### Patients

The institutional review board approved this retrospective study. The need for informed consent was waived due to the retrospective nature of this analysis and data anonymity. The clinical records were reviewed to identify patients for analysis.

The inclusion criterion was the patients who underwent [^123^I]-MIBG planar and SPECT/CT scan due to suspect of PHEO or PGL from April 2018 to March 2023. The exclusion criteria were as follows: (1) patients without pathologically diagnosed PHEO/PGL based on the assessment of surgical specimens, (2) patients with surgical specimens that were not graded using the PASS and GAPP, and (3) patients with head and neck PGLs because either PASS or GAPP scoring system was not applied.

**Fig. 1:**
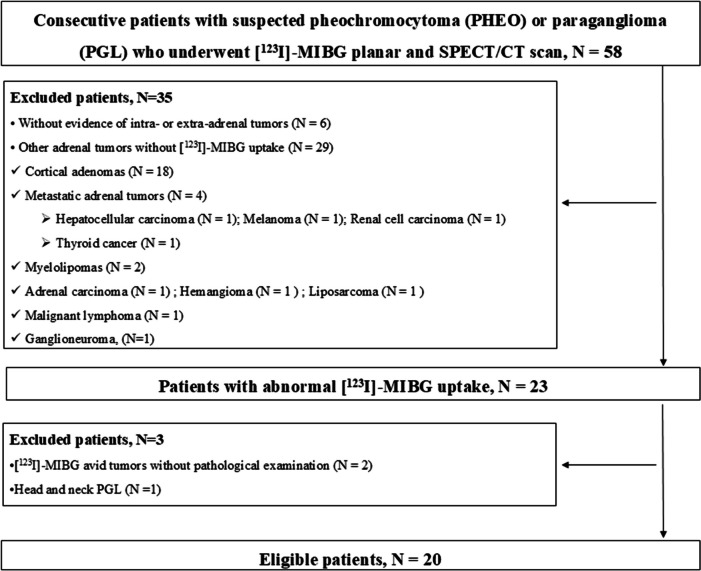
Flowchart of the study patient selection steps

From April 2018 to March 2023, [^123^I]-MIBG planar and SPECT/CT scan were performed on 58 consecutive patients with suspected PHEO or PGL. The flowchart of the study patient selection steps is shown in Fig. 1. Among them, 35 without PHEO/PGL were excluded from the analysis; 1) Six patients did not present with intra- or extra-adrenal tumors, and no abnormal [^123^I]-MIBG uptake was observed in all of them. 2) Twenty-nine had adrenal tumors other than PHEO/PGL (18 cortical adenomas; 4 metastatic adrenal tumors [one hepatocellular carcinoma, one melanoma, one renal cell carcinoma, and one thyroid cancer]; 2 myelolipomas, one adrenal carcinoma, one hemangioma, one liposarcoma, one malignant lymphoma, and one ganglioneuroma), and no [^123^I]-MIBG uptake was observed in all of them. Two patients with [^123^I]-MIBG avid adrenal tumors who did not undergo pathological examination were excluded. One patient with head and neck PGL was excluded because neither the PASS nor GAPP scoring system was applied in the assessment.

Finally, 20 (13 men, 7 women; mean [± SD] age: 54 ± 17 [range: 23–76] years) patients were eligible for the analyses. The patient’s medication status was also evaluated because some drugs, such as antihypertensive calcium-channel blockers, can reduce [^123^I]-MIBG uptake [15–17]. Plasma catecholamine concentrations were measured before surgery and within 3 months after [^123^I]-MIBG imaging (mean ± standard deviation [SD]: 22 ± 20 days; range: − 62 to + 66 days).

We previously examined whether the SUVmax of either myocardial or adrenal [^123^I]-MIBG can characterize myocardial function in patients with PHEO [18]. However, analyses of the correlation between [^123^I]-MIBG adrenal uptake and pathological grading in PHEOs or PGLs were not performed in the previous study. Thus, the purpose of this current study is different from the previous study [18], and the current study enrolled 18 former examined patients with PHEO who underwent 18 [^123^I]-MIBG planar and SPET/CT scans between April 2018 and August 2021.

### Imaging Protocols for [^123^I]-MIBG SPECT/CT Scan

The planar images of the anterior and posterior views and chest to abdomen (n = 18) or abdomen to pelvis (n = 2) SPECT/CT scan images were collected at 1 day after the intravenous injection of [^123^I]-MIBG at a dose of 111 MBq (3 mCi) (PDR Pharma, Japan) using a dual-head gamma camera with medium-energy parallel-hole collimators and a multidetector (16-row) spiral CT scan (Siemens Intevo SPECT/CT system; Siemens Medical Solutions USA Inc.). Planar images were acquired with a 256 × 1024 matrix, and the photopeak energy window was 159 (± 15%) keV for [^123^I]. SPECT/CT scan images were acquired with a matrix size of 128 × 128. A total image with 30 frames was acquired with an acquisition time of 30 s/frame and an angular step of 6°. After SPECT acquisition, CT scan images were acquired using a tube voltage of 130 kV and a dose-modulation algorithm with a quality reference mAs setting of 15 (CAREDose 4D; Siemens Medical Solutions USA Inc.). SPECT data were reconstructed with attenuation and scatter correction using a three-dimensional iterative algorithm (Ordered Subset Conjugate-Gradient Minimizer; Siemens Medical Solutions USA Inc.).

### Analysis of [^123^I]-MIBG Images

The [^123^I]-MIBG planar, SPECT, and SPECT/CT scan images were displayed on a workstation (Syngo.via; Siemens Healthcare GmbH, Erlangen, Germany; Advantage Windows Workstation 4.5; GE Healthcare, Milwaukee, WI) and were reviewed by two radiologists who were knowledgeable about the study’s purpose but who were blinded to the patients’ clinical information. The readers independently assessed the tumor [^123^I]-MIBG uptake on the planar images using the following score scales: uptake score of 0, no uptake; uptake score of 1, faint uptake that is less than that in the liver; uptake score of 2, uptake equal to that in the liver; and uptake score of 3, uptake greater than that in the liver [19]. These independent uptake scores were used to examine interobserver variability. Disagreements were resolved via a consensus decision between the two readers. The consensus uptake scores were used for the quantitative analyses. To determine the presence or absence of [^123^I]-MIBG uptake in the PHEO/PGL, scores of 0–1 and 2–3 were nonvisible and visible, respectively.

The following semi-quantitative analyses according to the interpreted results of the visual assessment were performed using the dedicated software (Syngo.via; Siemens Healthcare GmbH, Erlangen, Germany) by the abovementioned two readers independently. A previous study has shown the phantom experimental method used to calculate cross-calibration factor for converting SPECT count images to SPECT SUV images [18]. The SUV-related parameters of PHEO/PLG were generated as follows: First, the volume of interest (VOI) was drawn manually around the PHEO/PLG in a suitable reference transaxial plane, excluding the adjacent avid non-PHEO/non-PGL structures using the CT scan images as reference. We defined the SUVmax as the maximum tissue concentration in the structure delineated by the VOI divided by the activity injected per gram of body weight. Next, a threshold of 40% SUVmax was set to automatically delineate the VOI that met or exceeded this threshold. This VOI was used to calculate the mean SUV (SUVmean), tumor volume of [^123^I]-MIBG uptake (TV_MIBG), and total lesion [^123^I]-MIBG uptake (TL_MIBG). The TL_MIBG was calculated as the SUVmean multiplied by the TV_MIBG.

The averaged values of these SUV-related parameters obtained by the two readers were used for quantitative analyses.

### Pathological Examination and Grading

PHEO or PGL was confirmed via pathological examination in all cases. Hematoxylin and eosin staining and Ki-67 (DAKO, M7240) immunohistochemistry (IHC) staining were performed on a single representative block for each tumor. For the Ki-67 labeling index (%), two of the most highly labeled areas (hot fields, magnification of × 200) were counted by two pathologists.

An experienced pathologist who was blinded to the patients’ clinical information assessed PASS based on the published criteria and the histopathologic criteria for the GAPP score including Ki-67 staining (Supplemental Table 1). The PASS score incorporates 12 histologic parameters (large cell nests/diffuse growth, central necrosis, high cellularity, cellular monotony, tumor cell spindling, high mitotic index [> 3/10 high power fields], atypical mitosis, extension into adipose tissue, vascular invasion, capsular invasion, profound nuclear pleomorphism, and nuclear hyperchromasia) [2]. The PASS scores (range: 1–20) were categorized as < 4 and ≥ 4. PHEO or PGL with a PASS score of ≥ 4 was considered as biologically aggressive [2, 4]. Meanwhile, those with a PASS score of < 4 had a non-metastatic potential [2, 4]. The GAPP grading system was presented by 6 parameters which including 4 histological features (histological pattern, cellularity, coagulation necrosis, capsular/vascular invasion), immunohistochemical (Ki67 labelling index) and biochemical features (catecholamine type) [3]. The final GAPP score was established by incorporating data on dominant tumors secreting catecholamine. Tumors with significantly high plasma or urinary epinephrine levels with or without elevated norepinephrine levels were classified as epinephrine-secreting tumors. These tumors were assigned with a score of 0. Tumors with high norepinephrine levels without elevated epinephrine levels, with or without elevated dopamine levels were classified as norepinephrine-secreting tumors. These tumors were assigned with a score of 1. The nonfunctioning type was assigned with a score of 0. Based on the published cutoff points for risk stratification, the GAPP score was categorized as follows: 0–2, well-differentiated; 3–6, moderately differentiated; and 7–10, poorly differentiated [3]. In our institution, routine genetic testing such as assessment of succinate dehydrogenase subunit B (SDHB) mutation for PHEOs/PGLs was not performed.

### Statistical Analysis

The interobserver agreement of the uptake scoring was evaluated using κ statistics analysis. κ was interpreted as follows: < 0.20, slight agreement; 0.21–0.40, fair agreement; 0.41–0.60, moderate agreement; 0.61–0.80, substantial agreement; and ≥ 0.81, almost perfect agreement [20]. The Mann–Whitney U test or the Fisher’s exact test was used to assess the difference between two quantitative variables or to compare categorical data. The Spearman’s rank correlation was used to assess the association between two quantitative variables.

Data were expressed as median, interquartile range (IQR), and/or range. A *p* value of < 0.05 was considered statistically significant, and all *p*-values were two-sided. Statistical analyses were performed using MedCalc (MedCalc Software Ltd., Mariakerke, Belgium).

## Results

### Characteristics of the Patients

Table 1 shows the characteristics of 20 patients. Further, 18 patients were diagnosed with PHEOs and 2 with PGLs. In particular, six patients presented with dominant epinephrine-secreting tumors, 13 with dominant norepinephrine-secreting tumors, and 1 with nonfunctioning tumor. The Ki-67 indexes were < 1% in 10 tumors and 1%–3% in 10 tumors. The median PASS and GAPP scores were 2.0 (IQR: 0.0–3.0, range: 0.0–9.0) and 2.0 (IQR: 2.0–3.0, range: 1.0–6.0), respectively. In total, 16 (80%) and 4 tumors had a PASS score of < 4 and ≥ 4, respectively. According to the GAPP score, 11 and 9 tumors were well- and moderately differentiated, respectively.

**Table 1:** Characteristics of patients with pheochromocytoma/paraganglioma (N = 20)

Characteristics	Number		
Age (years) (mean, range)	54, 23–76		
Sex			
Male	13		
Female	7		
Tumor type			
Pheochromocytoma	18		
Paraganglioma	2		
Ki-67 immunoreactivity			
> 3%	0		
1–3%	10		
< 1%	10		
Dominant secreted catecholamine			
Epinephrine	6		
Norepinephrine	13		
Nonfunctioning	1		
PASS	Median	IQR	Range
	2.0	0.0–3.0	0.0–9.0
	Number		
PASS score of < 4	16		
PASS score of ≥ 4	4		
GAPP	Median	IQR	Range
	2.0	2.0–3.0	1.0–6.0
	Number		
Well-differentiated	11		
Moderately differentiated	9		
Poorly differentiated	0		

*PASS* Pheochromocytoma of the Adrenal Gland Scaled Score, *GAPP* Grading System for Adrenal Pheochromocytoma and Paraganglioma, *IQR* interquartile range

None of the patients received drugs that can reduce [^123^I]-MIBG uptake, except for antihypertensive calcium-channel blocker [15–17]. All patients were treated with antihypertensive drugs. Moreover, 12 patients received alpha-blockers; 4, calcium-channel blockers; 2, alpha-blockers and calcium-channel blockers; 1, angiotensin II receptor blockers; and 1, diuretics.

### Association between PHEO/PGL [^123^I]-MIBG Uptake and the PASS and GAPP Scores

All PHEOs and PGLs were visualized on [^123^I]-MIBG scintigraphy (median uptake score: 3.0, IQR: 2.0–3.0, range: 2.0–3.0). The uptake scores and SUV-related parameters did not significantly differ between tumors with a PASS score of < 4 and those with a PASS score of ≥ 4 in patients with PHEOs/PGLs (Table 2) (each, *p* > 0.05).

**Table 2:** Uptake scores and SUV-related parameters between tumors with a PASS score of < 4 and those with a PASS score of ≥ 4 in patients with pheochromocytoma/paraganglioma

Parameters	Tumors with a PASS score of < 4 (n = 16)			Tumors with a PASS score of ≥ 4 (n = 4)			*p* value
	Median	IQR	Range	Median	IQR	Range	
Uptake score	3.0	2.0–3.0	2.0–3.0	3.0	3.0–3.0	3.0–3.0	0.11
SUVmax	9.03	5.26–20.7	2.37–45.69	13.83	8.89–52.18	8.14–86.23	0.49
SUVmean	6.22	3.42–11.88	1.73–26.05	7.95	5.21–30.77	4.65–51.43	0.49
TV_MIBG (mL)	46.78	41.95–68.25	29.60–256.63	69.19	57.09–85.13	48.58–97.48	0.10
TL_MIBG	304.80	165.45–662.57	72.26–6684.39	701.33	362.38–1740.58	304.66–2498.61	0.18

*PASS* Pheochromocytoma of the Adrenal Gland Scaled Score, *IQR* interquartile range, *TV_MIBG* tumor volume of [^123^I]-MIBG uptake, *TL_MIBG* total lesion [^123^I]-MIBG uptake

Table 3 shows the uptake score and SUV-related parameters between well- and moderately differentiated PHEOs/PGLs. Moderately differentiated tumors had a significantly higher uptake score than well-differentiated tumors (*p* = 0.048). Moderately differentiated tumors had a significantly higher SUVmax and SUVmean than well-differentiated tumors (*p* = 0.001 and 0.007, respectively). Moderately differentiated tumors had a significantly higher TL_MIBG than well-differentiated tumors (*p* = 0.031). However, there was no significant difference in TV_MIBG between well- and moderately differentiated tumors (*p* = 0.88).

**Table 3:** Uptake scores and SUV-related parameters between well-differentiated tumors and moderately differentiated tumors in patients with pheochromocytoma/paraganglioma

Parameters	Patients with well-differentiated tumors (n = 11)			Patients with moderately differentiated tumors (n = 9)			*p* value
	Median	IQR	Range	Median	IQR	Range	
Uptake score	2.0	2.0–3.0	2.0–3.0	3.0	3.0–3.0	2.0–3.0	0.048
SUVmax	8.14	4.46–9.31	2.37–32.49	18.99	13.98–29.17	5.71–86.23	0.001
SUVmean	4.65	2.80–6.63	1.73–17.91	11.34	8.10–17.13	4.18–51.43	0.007
TV_MIBG (mL)	56.51	45.43–66.51	41.87–97.48	48.58	38.21–72.17	29.60–256.63	0.88
TL_MIBG	244.79	151.19–440.02	72.26–982.55	521.80	342.94–1374.72	149.71–6684.39	0.031

*IQR* interquartile range, *TV_MIBG* tumor volume of [^123^I]-MIBG uptake, *TL_MIBG* total lesion [^123^I]-MIBG uptake

As shown in Table 4, a significant positive correlation was observed between the uptake score and PASS scores (ρ = 0.50, *p* = 0.023). However, there were no significant correlations between the SUV-related parameters and PASS score (each, *p* > 0.05). By contrast, significant positive correlations were observed between the GAPP score and the uptake score (ρ = 0.62, *p* = 0.003), SUVmax (ρ = 0.63, *p* = 0.003), SUVmean (ρ = 0.63, *p* = 0.003), and TL_MIBG (ρ = 0.57, *p* = 0.009). However, no significant correlation was observed between the GAPP score and TV_MIBG (ρ = 0.10, *p* = 0.68).

**Table 4:** Correlations of PASS and GAPP scores with uptake scores and SUV-related parameters in patients with pheochromocytoma/paraganglioma

Parameter	PASS scores		GAPP scores	
	Correlations		Correlations	
	ρ	*p* value	ρ	*p* value
Uptake score	0.50	0.023	0.62	0.003
SUVmax	0.33	0.16	0.63	0.003
SUVmean	0.33	0.16	0.63	0.003
TV_MIBG	0.22	0.35	0.10	0.68
TL_MIBG	0.40	0.078	0.57	0.009

*PASS* Pheochromocytoma of the Adrenal Gland Scaled Score, *GAPP* Grading System for Adrenal Pheochromocytoma and Paraganglioma, *TV_MIBG* tumor volume of [^123^I]-MIBG uptake, *TL_MIBG* total lesion [^123^I]-MIBG uptake

The uptake scores and any SUV-related parameters did not significantly differ between patients treated with calcium-channel blockers and those who were not (each, *p* > 0.05; Supplemental Table 2).

**Fig. 2:**
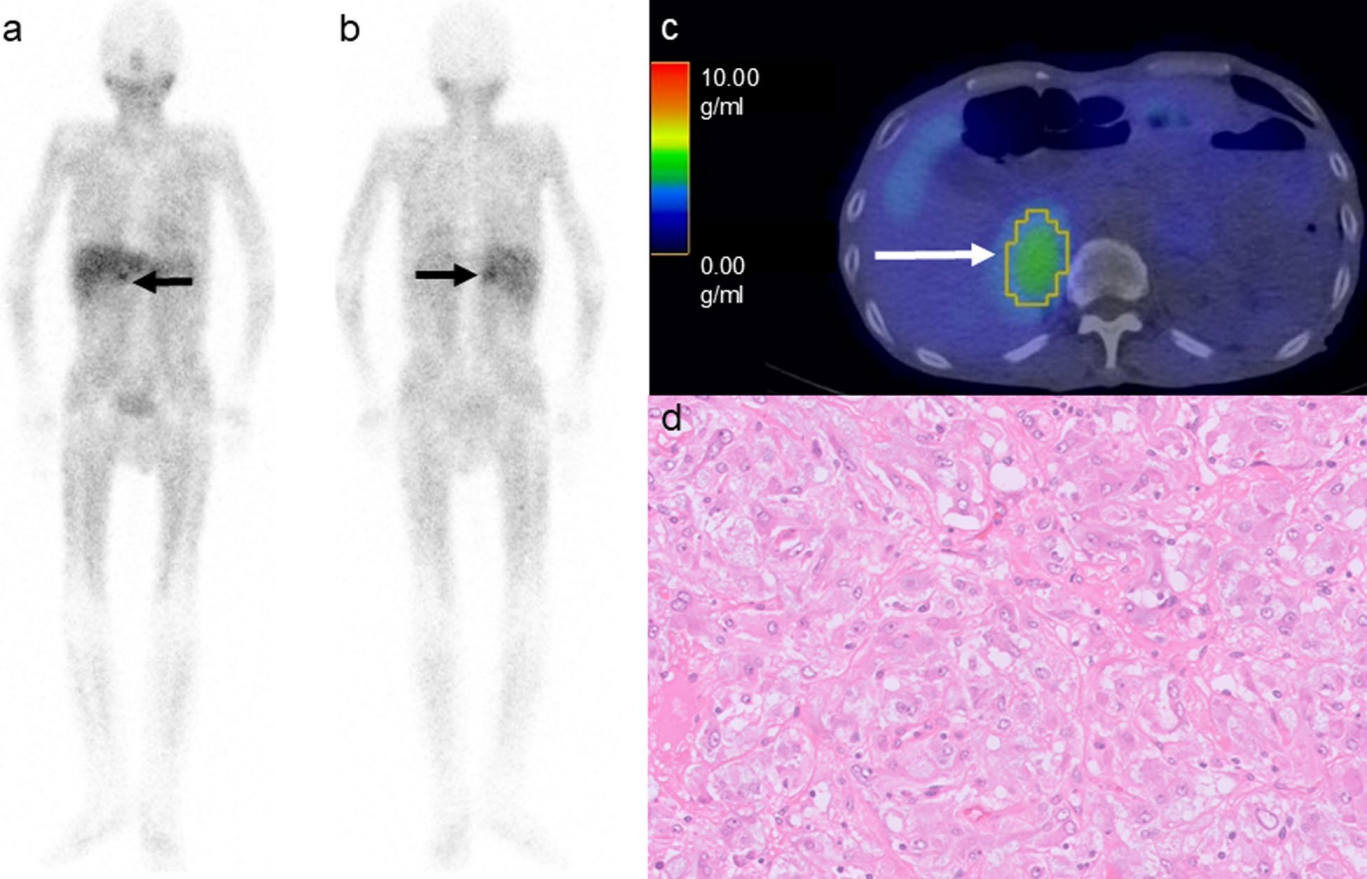
A 76-year-old male patient with right adrenal pheochromocytoma. The anterior (**a**) and posterior (**b**) [^123^I]-MIBG planar images showing uptake in the pheochromocytoma (arrows, score of 2). The [^123^I]-MIBG SPECT/CT scan images showing the [^123^I]-MIBG uptake in the pheochromocytoma (**c**): arrow). The SUVmax, SUVmean, TV_MIBG, and TL_MIBG were 4.34, 3.05, 46.29 mL, and 141.19, respectively. Microphotograph (**d**: Hematoxylin & Eosin staining, × 200) showing a regular Zellballen pattern with a low cellularity. The PASS and GAPP scores were 0 and 1, respectively. According to the GAPP score, this was as a well-differentiated tumor

**Fig. 3:**
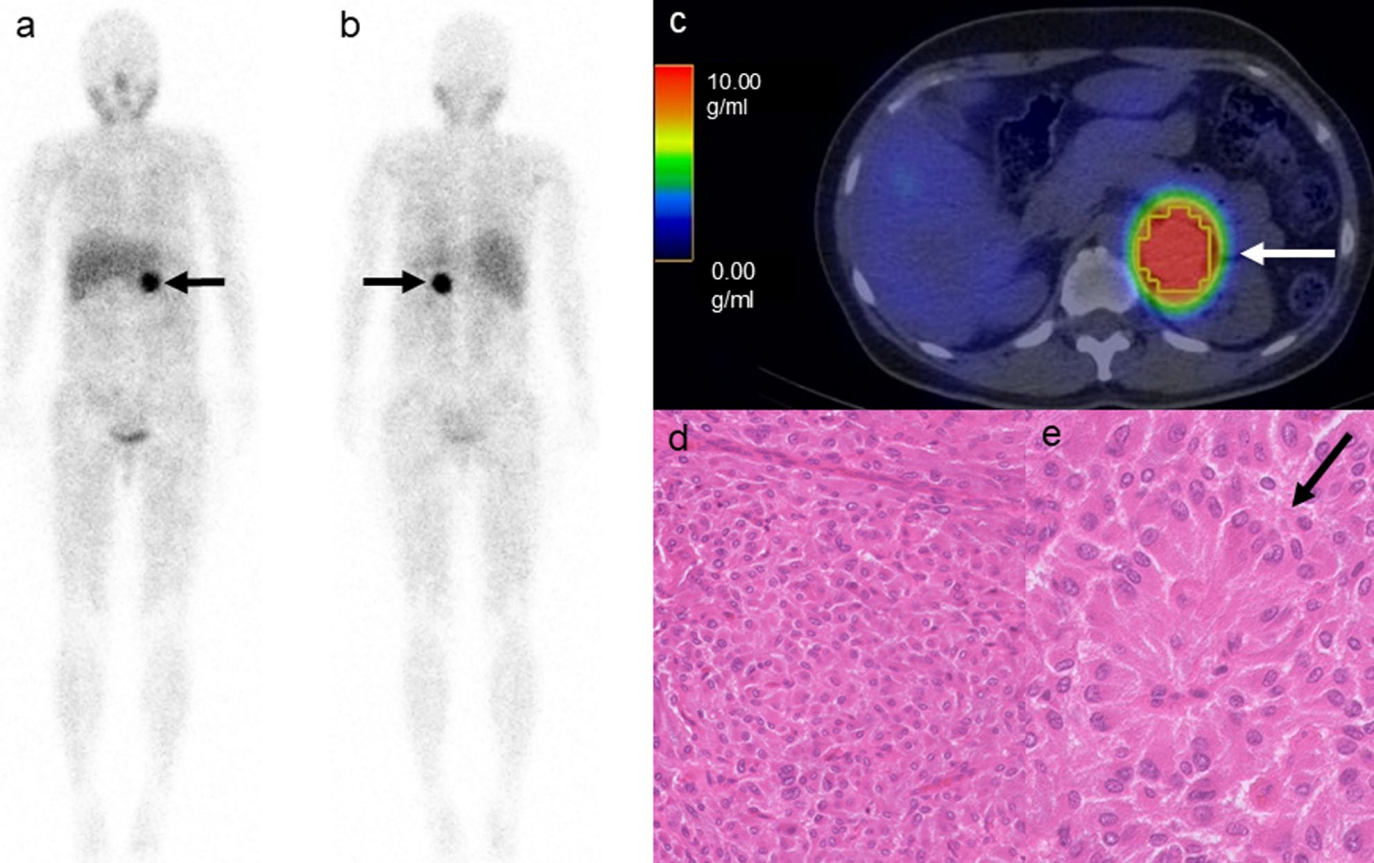
A 50-year-old female patient with left adrenal pheochromocytoma. The anterior (**a**) and posterior (**b**) [^123^I]-MIBG planar images showing an intense pheochromocytoma uptake (arrows, score of 3). The [^123^I]-MIBG SPECT/CT scan images showing the [^123^I]-MIBG uptake in the pheochromocytoma (**c**: arrow). The SUVmax, SUVmean, TV_MIBG, and TL_MIBG were 22.44, 12.41, 42.04 mL, and 521.80, respectively. Microphotographs (**d**, **e**: Hematoxylin & Eosin staining) showing a large irregular Zellballen pattern with a high cellularity (**d**: × 200) and pseudorosette pattern (**e**: arrow, × 400). The PASS and GAPP scores were 1 and 6, respectively. According to the GAPP score, this was a moderately differentiated tumor

**Fig. 4:**
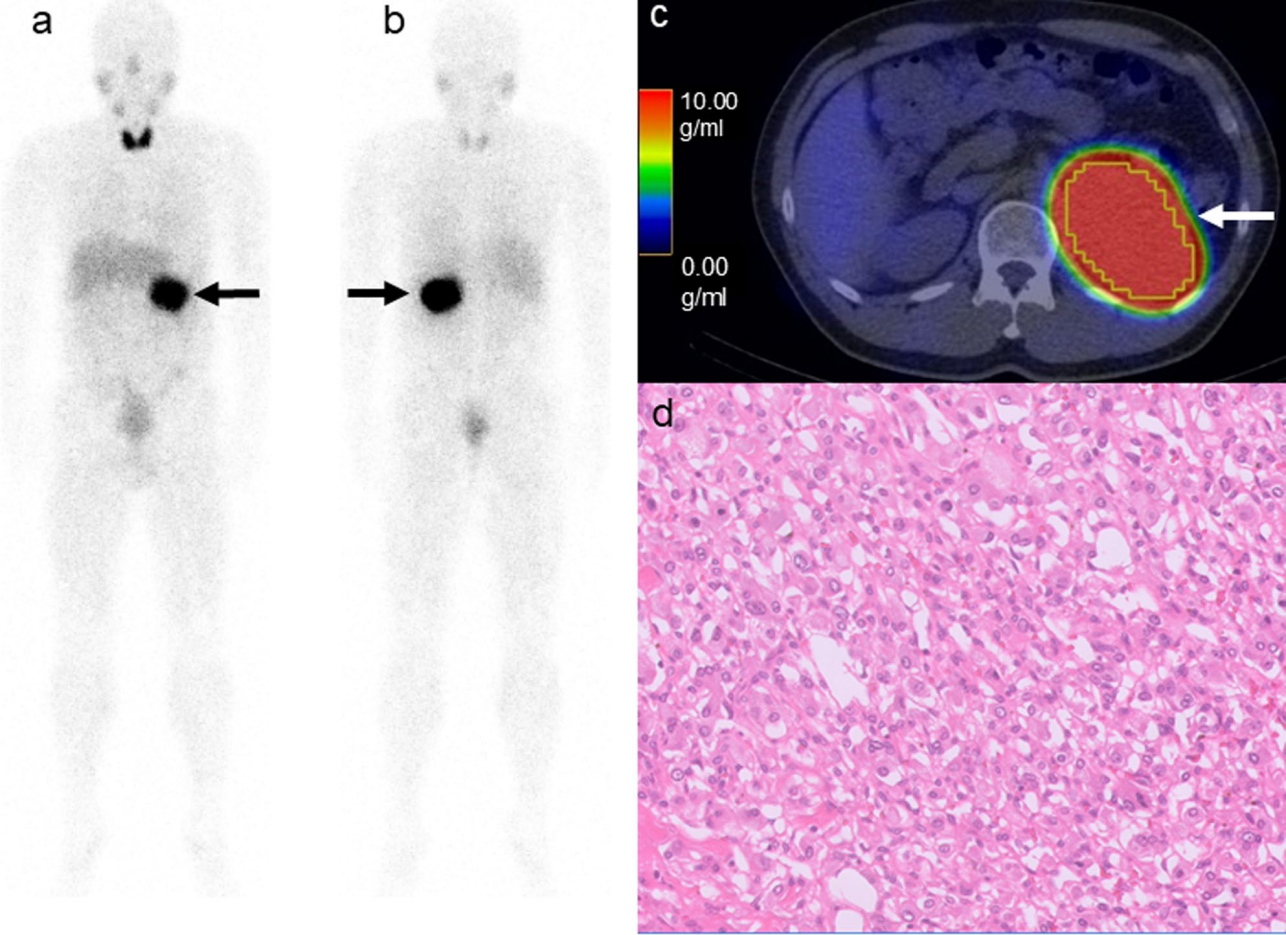
A 42-year-old male patient with left adrenal pheochromocytoma.The anterior (**a**) and posterior (**b**) [^123^I]-MIBG planar images showing an intense pheochromocytoma uptake (arrows, score of 3). The [^123^I]-MIBG SPECT/CT scan images showing the [^123^I]-MIBG uptake in the pheochromocytoma (**c**: arrow). The SUVmax, SUVmean, TV_MIBG, and TL_MIBG were 52.03, 30.15, 160.62 mL, and 4843.15, respectively. Microphotograph (**d**: Hematoxylin & Eosin staining, × 200) showing a large irregular Zellballen pattern with a moderate cellularity. The PASS and GAPP scores were 6 and 3, respectively. According to the GAPP score, this was a moderately differentiated tumor

Figures 2, 3 and 4 show the representative [^123^I]-MIBG images of PHEO/PGLs with the PASS and GAPP scores.

### Interobserver Variability

Supplemental Table 3 shows the interobserver agreements for uptake score, SUVmax, SUVmean, TV_MIBG, and TL_MIBG. The interobserver agreement for SUVmax between two readers was perfect. The interobserver agreements for uptake score, SUVmean, TV_MIBG, and TL_MIBG between two readers were almost perfect, ranging from 0.81 (uptake score: 95% confidence intervals [CIs] = 0.57–1.00) to 0.95 (SUVmean: 95% CIs = 0.93–0.98).

## Discussion

The current study evaluated the potential usefulness of [^123^I]-MIBG SUV-related parameters for characterizing the metastatic potential in patients with PHEO/PGL. In the current study, there were no significant correlations between PASS and SUV-related parameters. However, the scores of the GAPP, which is a newly proposed system for assessing the metastatic potential of PHEO/PGL, were positively related to [^123^I]-MIBG uptake, which is assessed using SUVmax, SUVmean, and TL_MIBG. Therefore, the tumor [^123^I]-MIBG uptake, assessed with SUV-related parameters, could potentially be a tool for non-invasive imaging to characterize the metastatic potential in patients with PHEO/PGL.

Two major pathological grading systems have been established to assess the metastatic risk of PHEO and PGL: the PASS and GAPP grading systems. Developed by Thompson in 2002, the PASS grading system was the first to evaluate the likelihood of aggressive biological behavior in PHEO [2]. It evaluates 12 distinct histological features, which together make up a score of 20, as detailed in Supplemental Table 1. According to Thompson’s PASS scoring criteria, lesions with a score of 4 or higher are predicted to have a higher risk of metastasis, whereas those scoring below 4 are deemed to have a lower risk [2]. A recent meta-analysis reported that using a PASS cutoff of ≥ 4, the sensitivity, specificity, positive predictive value, and negative predictive value for predicting metastatic PHEO were 97%, 68%, 31%, and 99%, respectively [21]. This suggests that the system is more reliable at identifying non-metastatic cases than metastatic ones. The GAPP grading system, introduced by Kimura et al. in 2014, evaluates PHEOs/PGLs based on four histological criteria, one immunohistochemical marker, and one biochemical marker, all detailed in the Supplemental Table  1 , giving a maximum score of 10 [3]. Tumors are categorized into three grades: well-differentiated (0–2 points), moderately differentiated (3–6 points), and poorly differentiated (7–10 points). This system was developed to assess its effectiveness in predicting the likelihood of metastasis and patient outcomes for PHEO/PGL cases. Their research indicated that well-differentiated tumors had a low metastasis rate of 4% and a 100% five-year survival rate, whereas the moderately differentiated tumors had a 60% metastasis rate with a 67% five-year survival rate, and poorly differentiated tumors showed an 88% metastasis rate with only a 22% five-year survival rate [3]. These results suggest that well-differentiated tumors present a low metastatic risk, whereas moderately and poorly differentiated tumors are more prone to metastasize, especially with a GAPP score of 3 or higher. Therefore, the GAPP grading system potentially aids in estimating both metastatic risk and prognosis based on tumor scores and differentiation levels [3]. Despite their utility, these grading systems have some limitations. The PASS system relies solely on histological characteristics, which can be numerous and challenging to evaluate [5]. Although the GAPP system was developed to refine PASS by removing certain non-specific elements and incorporating factors like Ki-67 index and catecholamine type, it still omits critical aspects such as genetic mutations and tumor location, which are known to influence metastasis and patient prognosis [5]. Additionally, these grading systems have not been extensively validated through multicenter clinical trials and are not yet widely endorsed by clinicians [5].

The uptake and retention of MIBG are facilitated by the norepinephrine transporter system present in the plasma membranes of sympathetic neurons, ganglia, and chromaffin cells [22, 23], along with intracellular vesicular monoamine transporters [24]. Due to this uptake mechanism, [^123^I]-MIBG scintigraphy is widely used as a functional imaging method to detect PHEO/PGL originating from chromaffin cells [6–8]. A meta-analysis confirmed the effectiveness of [^123^I]-MIBG scintigraphy for identifying PHEO, with reported sensitivity and specificity rates of 94% and 92%, respectively [25]. Although imaging-guided biopsies are employed for differential diagnoses, biopsies of suspected PHEO/PGL are discouraged because of risks like hemorrhage or hypertensive crisis, necessitating surgical excision instead [26]. Other meta-analysis indicated that the sensitivity of [^123^I]-MIBG scintigraphy is 79% in patients with metastatic PHEO/PGL and 96% in those with non-metastatic forms [27]. A prospective multicenter study evaluating [^123^I]-MIBG scintigraphy for PHEO/PGL diagnosis reported a sensitivity of 86% for primary lesions and 83% for metastatic diseases [28]. Thus, metastatic PHEOs/PGLs were more likely to be false-negative compared with nonmetastatic PHEOs/PGLs on [^123^I]-MIBG scintigraphy [9]. Previous studies have shown that de-differentiation is associated with [^123^I]-MIBG scintigraphy-negative PHEOs/PGLs [10, 27].

On the contrary, our results showed that not only the uptake score but also the semi-quantitative parameters including SUVmax, SUVmean, and TL_MIBG were significantly higher in moderately differentiated tumors than in well-differentiated tumors. Moreover, there were significant positive correlations between the uptake scores and GAPP scores, and between the SUV-related parameters and GAPP scores except TV_MIBG. Our results were inconsistent with those of the abovementioned studies [9, 10]. Previous studies have revealed that the [^123^I]-MIBG uptake might be negatively correlated with the differentiation of PHEOs/PGLs. However, the abovementioned previous studies examined the differences in the [^123^I]-MIBG uptake of primary or metastatic PHEOs/PGLs assessed via visual assessment only, and the semi-quantitative analyses of [^123^I]-MIBG uptake were not performed [9, 10]. To the best of our knowledge, no study has previously investigated the association between the [^123^I]-MIBG uptake assessed via semi-quantitative analysis using SUV-related parameters and pathological differentiation in PHEOs/PGLs. Bomanji et al. [11] reported that one patient with metastatic PHEO (0.13%) and one patient with metastatic PGL (0.14%) had a higher [^123^I]-MIBG uptake calculated based on the injected dose per gram of tissues than two patients with nonmetastatic PHEOs (each, 0.01%) and one patient with nonmetastatic PGL (0.02%). Maurea et al. [12] revealed that the [^131^I]-MIBG uptake calculated using intensity ratio (tumor/normal tissue) in metastatic PHEOs/PGLs was significantly higher than that in nonmetastatic PHEOs/PGLs (*p* < 0.03). Although metastases were not observed at the time of patient inclusion in our study, our results are in accordance with those of previous reports. MIBG uptake reflected the concentration of neurosecretory storage in PHEOs/PGLs. Hence, these previous studies hypothesized that the higher MIBG uptake observed in metastatic PHEOs/PGLs might reflect a greater concentration of stored catecholamine.

In examining SUV-related parameters, no significant connection was found between TV_MIBG (tumor volume of [^123^I]-MIBG uptake) and the GAPP grading system: The TV_MIBG values were not significantly different across well- to moderately differentiated tumors, nor did they correlate significantly with GAPP scores. According to Agarwal et al. [29], there is no evident correlation between tumor size and the PASS grading system, which is a notable pathological grading method as mentioned above. Our findings also indicate no notable association between TV_MIBG and the PASS grading system. Therefore, it is possible that the tumor volume of [^123^I]-MIBG uptake does not correlate with existing pathological grading systems. Further studies should be conducted to explore the relationship between TV_MIBG and the PASS or GAPP grading systems.

In our study, a discrepancy was observed in the association between the [^123^I]-MIBG uptake between the PASS and GAPP grading systems. The GAPP score was positively related to [^123^I]-MIBG uptake. However, no correlations were observed between the PASS and [^123^I]-MIBG uptake. Although the cause of this difference is unknown, the different characteristics between the PASS and GAPP scoring systems might have been affected. As mentioned above, the PASS system only incorporates histologic features and other important factors such as the immunohistochemical (Ki-67 index) and clinical characteristics (catecholamine type) of patients were not included [5, 21]. By contrast, the GAPP included some histological features from the PASS and added immunohistochemical (Ki-67 index) and biochemical (catecholamine type) data. Recently, Wachtel et al. [30] found that a higher GAPP score, but not the PASS score, was associated with metastatic PHEOs/PGLs. Moreover, they recommended a shift from PASS to GAPP scores to predict a more accurate prognostication for patients with PHEOs/PGLs.

This study had several limitations that should be discussed. First, its sample size was extremely small. Hence, a definitive conclusion could not be obtained, and prospective studies with a larger study population should be further performed. Second, the number of PHEO cases was larger than PGL cases in the study population. This could be due to the influence of data from a previous study focused on PHEOs, potentially leading to biased numbers. However, PHEOs are generally more common than PGLs, making up between 78.4% and 95.8% of all PHEO/PGL instances [31]. Third, the association between genetic mutations and [^123^I]-MIBG uptake was not examined as genetic testing was not routinely performed at our institution. Fourth, tumor [^123^I]-MIBG uptake could have been reduced in patients receiving calcium-channel blockers. However, neither the uptake score nor the SUV-related parameters differed significantly between patients treated with calcium-channel blockers and those who were not. Fifth, only patients with well- and moderately differentiated PHEOs/PGLs were included in the analysis. De-differentiation could decrease the norepinephrine transporters and adrenergic storage vesicles of PHEOs/PGLs [10]. Thus, further studies should be performed to explore the definite association between [^123^I]-MIBG uptake and the differentiation of PHEOs/PGLs in a larger study population including those with poorly differentiated PHEOs/PGLs. Finaly, only the association between [^123^I]-MIBG uptake and pathological grading systems, not that between [^123^I]-MIBG uptake and patient prognosis, was examined. Indeed, none of the patients presented with recurrence or distant metastasis at the final follow-up, which was in July 2023.

## Conclusion

The primary tumor [^123^I]-MIBG uptake assessed using SUV-related parameters can be an imaging tool for predicting metastasis in patients with PHEO/PGL.

## Supplementary Information

Below is the link to the electronic supplementary material.

Supplementary file1 (DOCX 43.5 KB)

## Data Availability

The data that support the findings of this study are available from the corresponding author upon reasonable request.
