# Supplementary material for: Usefulness of the Primary Tumor Standardized Uptake Value of Iodine-123 Metaiodobenzylguanidine for Predicting Metastatic Potential in Pheochromocytoma and Paraganglioma
Source: Mol Imaging Biol. 2024 Sep 18;26(6):1005–15. doi: 10.1007/s11307-024-01952-8 (PMC11635048; doi:10.1007/s11307-024-01952-8)
Supplement: Supplementary file 1 — Supplementary file1 (DOCX 43.5 KB) [file 11307_2024_1952_MOESM1_ESM.docx]

| **Supplemental Table 1.** Pheochromocytoma of the Adrenal Gland Scaled Score (PASS) and Grading System for Adrenal Pheochromocytoma and Paraganglioma (GAPP) | |
| --- | --- |
| Characteristics | Scores |
| PASS |  |
| Large nests or diffuse growth | 2 |
| Central or confluent tumor necrosis | 2 |
| High cellularity | 2 |
| Cellular monotony | 2 |
| Tumor cell spindling | 2 |
| Mitotic figures of >3/10 HPF | 2 |
| Atypical mitotic figure(s) | 2 |
| Extension into the adipose tissues | 2 |
| Vascular invasion | 1 |
| Capsular invasion | 1 |
| Profound nuclear pleomorphism | 1 |
| Nuclear hyperchromasia | 1 |
| Maximum score | 20 |
|  |  |
| GAPP |  |
| Pattern |  |
| Zellballen | 0 |
| Large and irregular-sized nest | 1 |
| Pseudorosette formation | 1 |
| Cellularity |  |
| Low (<150/62.5 mm^2^) | 0 |
| Moderate (150–250/62.5 mm^2^) | 1 |
| High (>250/62.5 mm^2^) | 2 |
| Coagulation necrosis |  |
| Presence | 2 |
| Absence | 0 |
| Vascular/capsular invasion |  |
| Presence | 2 |
| Absence | 0 |
| Ki-67 immunoreactivity |  |
| >3% or 50 cells/MPF | 2 |
| >1% or 20 cells/MPF | 1 |
| Few cells | 0 |
| Types of catecholamine |  |
| Norepinephrine | 1 |
| Epinephrine | 0 |
| Nonfunctioning | 0 |
| Maximum score | 10 |
| *HPF*, high-power field; *MPF*, medium-power field | |

| **Supplemental Table 2.** Uptake scores and SUV-related parameters between patients with pheochromocytoma/paraganglioma treated who were with calcium-channel blockers and those who were not | | | | | | | |
| --- | --- | --- | --- | --- | --- | --- | --- |
| Parameters | Patients treated with calcium-channel blockers (n = 6) | | | Patients who were not treated with calcium-channel blockers (n = 14) | | | *p* value |
|  | Median | IQR | Range | Median | IQR | Range |  |
| Uptake score | 3.0 | 3.0–3.0 | 3.0–3.0 | 3.0 | 2.0–3.0 | 2.0–3.0 | 0.11 |
| SUVmax | 11.96 | 8.29–17.23 | 8.14–18.99 | 9.57 | 5.26–23.05 | 2.37–86.23 | 0.96 |
| SUVmean | 7.15 | 5.03–10.11 | 4.65–11.34 | 6.40 | 3.42–13.28 | 1.73–51.43 | 0.96 |
| TV_MIBG (mL) | 42.04 | 34.27–55.37 | 29.60–65.59 | 57.45 | 45.44–71.31 | 36.07–256.63 | 0.10 |
| TL_MIBG | 320.21 | 274.73–340.55 | 244.79–345.33 | 455.29 | 165.49–914.61 | 72.26–6684.39 | 0.68 |
| *IQR*, interquartile range; *TV_MIBG*, tumor volume of [^123^I]-MIBG uptake; *TL_MIBG*, total lesion [^123^I]-MIBG uptake | | | | | | | |

| **Supplemental Table 3.** The κ values of uptake scores and SUV-related parameters for interobserver agreement between two readers | |
| --- | --- |
| Parameters | κ value |
| Uptake score | 0.81 (0.57–1.00) |
| SUVmax | 1.00 (1.00–1.00) |
| SUVmean | 0.95 (0.93–0.98) |
| TV_MIBG | 0.82 (0.63–1.00) |
| TL_MIBG | 0.94 (0.90–0.97) |
| ^a^ Numbers in parentheses are 95% confidence intervals. | |
